# Attitudes about sickness presenteeism in medical training: is there a hidden curriculum?

**DOI:** 10.1186/s13756-019-0602-7

**Published:** 2019-09-05

**Authors:** Lauris C. Kaldjian, Laura A. Shinkunas, Heather Schacht Reisinger, Marc A. Polacco, Eli N. Perencevich

**Affiliations:** 1 Department of Internal Medicine, Carver College of Medicine, University of Iowa, Iowa City, IA USA; 2 Program in Bioethics and Humanities, Carver College of Medicine, University of Iowa , 500 Newton Road, Iowa City, IA 52242 USA; 3 Center for Comprehensive Access and Delivery Research and Evaluation, Iowa City VA Health Care System, Iowa City, IA USA; 4 Department of Otolaryngology, Dartmouth-Hitchcock Medical Center, Lebanon, NH USA

**Keywords:** Sickness presenteeism, Infection control, Patient safety, Medical education, Hidden curriculum, Medical ethics

## Abstract

**Background:**

Sickness presenteeism among healthcare professionals can compromise patient safety. To better understand what motivates this phenomenon, especially among trainees, the authors investigated attitudes of medical students, resident physicians, and faculty physicians about working when sick with what might be an infectious condition.

**Methods:**

In 2012–2013, the authors employed a mixed methods, two-stage, cross-sectional survey at the University of Iowa Hospitals and Clinics of medical students (third-year students in the first survey and fourth-year students in the second survey), resident physicians in Internal Medicine, Pediatrics, and Family Medicine (first-year residents in the first survey and second-year residents in the second survey), and faculty physicians in Internal Medicine, Pediatrics, and Family Medicine. The first survey included one open-ended question querying attitudes about sickness presenteeism, answers to which underwent content analysis that identified 17 codes used to develop 23 additional closed-ended questions for a second survey.

**Results:**

127 participants completed the second survey (44% response rate). Sixty percent of these participants felt obligated to work when sick; and 33% felt obligated to work with influenza-like symptoms (fever, myalgias, cough), with residents and students being more likely to do so than faculty (67% vs. 35% vs. 14%, *p* = 0.001). Most participants (83%) were motivated to work when sick to avoid creating more work for colleagues, and residents and students were more likely than faculty physicians to want to avoid negative repercussions (84% vs 71% vs. 25%, *p* < 0.001) or appear lazy or weak (89% vs 75% vs. 40%, *p* < 0.001). Most participants also recognized the need to avoid spreading infections to patients (81%) or colleagues (75%).

**Conclusions:**

When deciding whether to work when sick, students, residents, and faculty report a mixture of motivations that focus on the interests of patients, colleagues, and themselves. Awareness of these mixed motivations, particularly among trainees, can help inform interventions aimed at limiting instances of sickness presenteeism to support a culture of patient safety and counter any tendencies toward a hidden curriculum of efficiency and achievement.

## Background

Sickness presenteeism is a concept that describes the actions of people who decide to work while they are sick [1–3]. It is particularly associated with the helping and teaching professions [1], due to the nature of the services provided and the needs of the persons served. Historically, the study of sickness presenteeism has focused on the economic impact of lost productivity caused by conditions or issues that prevent employees from being fully engaged in their work [4–6], though more recently there has also been a focus on the implications for employees’ own health [7].

In healthcare settings, sickness presenteeism raises important questions about risks to patient safety and the quality of care, especially in light of studies that demonstrate that large proportions (41–88%) of healthcare professionals across the globe report at least some practice of sickness presenteeism [8–17]. Moreover, some of these studies document the willingness of some healthcare professionals to work while they are sick with what is known or likely to be an infectious condition [9, 11–13, 16]. And the problem of sickness presenteeism appears to start early in the process of training, as demonstrated by five studies that have surveyed either medical students [18, 19] or resident physicians [18, 20–22] in the United States and Canada. These reports from training environments also suggest that there may be important differences in the reasons that motivate sickness presenteeism among students and residents, as compared with attending physicians – reasons that are directly related to trainees’ perceptions about work, learning, evaluation criteria, and academic success.

There is a double irony when sickness presenteeism occurs among healthcare professionals: not only does it indicate that such clinicians are disinclined to follow the advice they would presumably give to their own patients (time off from work while sick), but also that some clinicians appear to be willing to try to help patients while knowing that doing so (when they themselves are sick with an infectious condition) might actually cause harm. Based on the clinical-scientific understanding of the potential for healthcare professionals to serve as vectors of infectious diseases [23], coupled with established standards of ethics and professionalism that place priority on the primacy of patient welfare [24], this double irony needs to be discussed and addressed. And given the paucity of studies reporting data from training environments, there is a particular need for more understanding about trainees’ attitudes so that efforts to engage the problem of sickness presenteeism during training can be better informed and lead to enduring improvements in professional practice. To this end, we performed a two-part, cross-sectional survey of medical students, resident physicians, and faculty physicians to identify the attitudes that influence their decisions about whether or not to work when they are sick.

## Methods

### Study aim and design

We employed an anonymous, mixed methods, two-stage, cross-sectional survey in the autumn of 2012 and the autumn of 2013 to assess medical professionals’ attitudes toward issues related to infection control, including sickness presenteeism. The online survey was generated using Qualtrics software (Qualtrics Research Suite, Provo, Utah) and was estimated to require 5–10 min to complete. The study was approved and considered exempt by the University of Iowa Institutional Review Board.

### Setting

The University of Iowa Hospitals and Clinics is a comprehensive academic medical center and regional referral center in the US, with over 800 beds and more than 200 outpatient clinics and care areas.

### Study population

Three groups of medical professionals working at the University of Iowa Hospitals and Clinics were included: (1) medical students (third-year students in the first survey and fourth-year students in the second survey); (2) resident physicians in Internal Medicine, Pediatrics, and Family Medicine (first-year residents in the first survey and second-year residents in the second survey); (3) faculty physicians in Internal Medicine, Pediatrics, and Family Medicine. Four rounds of invitation emails were sent to potential participants with a link to the online survey, and a $10 token incentive for participation was provided. Different survey links were used for medical students, residents, and faculty, respectively, in order to tailor training-related questions to each participant group. The survey was anonymous and participation was not tracked, therefore we do not know how many respondents from the first survey may have participated in the second survey.

### Development of sickness presenteeism questions

The first survey included four closed-ended questions (using a 5-point Likert scale ranging from strongly agree to strongly disagree) and one open-ended question querying attitudes about sickness presenteeism. The open-ended question was: “When deciding whether or not to come to work when you are feeling ill, what is the primary factor you use in making the decision?” The first survey was completed by 147 participants (49% response rate), and 126 participants provided answers to the open-ended question. We coded the answers to these open-ended questions using a “conventional approach” to content analysis [25] to describe medical professionals’ attitudes toward sickness presenteeism and thereby develop new questions for the second survey. For coding, responses to the open-ended question were distributed to three members of the research team (LAS, LCK, HSR), who reviewed the responses independently for shared themes. Team members then met to develop a consensus-based codebook, which identified 27 items.

After an intentional two-week pause, the same three team members independently coded a fresh copy of the responses to the open-ended question using the 27-item codebook (a single response was allowed to receive more than one code, if needed). The results of this coding were compared for each code between two of the investigators (HSR and LAS), and for disagreements a third investigator acted as a tie breaker (LCK). Through this further iterative process, the number of codes was reduced from 27 to 17 by combining related themes into broader categories. For example, the codes “coverage”, “creating more work for my colleagues”, “hassle”, and “avoiding letting colleagues down” were combined into the single code (“creating more work for colleagues, including hassles related to coverage issues and letting ‘my team’ down”). After this process of refinement, the average kappa score for interrater reliability between two investigators (HSR and LAS) for these 17 codes was 0.869, and 12 codes demonstrated very good or perfect agreement (> 0.81), as shown in the Appendix.

For the second survey, these 17 codes were used to develop 23 additional closed-ended questions (using a 5-point Likert scale ranging from strongly agree to strongly disagree) that were added to the four closed-ended questions about sickness presenteeism carried over from the first survey. We did not ask any open-ended questions in the second survey. Some questions were training-level specific and therefore only administered to a specific participant group. Faculty physicians received 19 closed-ended questions about sickness presenteeism, and medical students and residents each received 21 closed-ended questions about sickness presenteeism. (Results from one question carried over from the first survey are omitted from this report because of its similarity to one of the questions added to the second survey.)

### Statistical analysis

Data from online surveys were downloaded into Microsoft Excel and imported into SAS statistical software (Cary, NC, version 9.3). Frequencies and chi-square tests were performed on data from the second survey to compare categorical data (dichotomized Likert scale responses) between participant groups. Not all questions were answered by every respondent in the second survey, and the largest number of non-responses for any one question was 7. Data in Tables 1, 2, and 3 represent percentages calculated using a denominator that excluded any missing data from the question involved.

**Table 1:** Attitudes about Working when Sick, Related to Severity and Type of Illness

	Agree or Strongly Agree				*P*-Value
	Totals	Students	Residents	Faculty	
I feel a professional obligation to come into work even when I am feeling sick.	60%	61%	84%	46%	0.02
*I would come into work with …*					
A cold (i.e., a mild upper respiratory infection without fever).	95%	96%	100%	92%	0.39
The flu (i.e., fever, myalgias, and cough).	33%	35%	67%	14%	0.001
A fever > 101.5.	13%	10%	37%	6%	0.003
A fever < 101.5.	59%	65%	68%	42%	0.046
Vomiting (1 or more episodes of vomiting within the last 8 h).	24%	15%	58%	22%	0.001
Diarrhea (3 or more watery stools within the last 8 h).	28%	25%	58%	17%	0.004

**Table 2:** Reasons for Coming into Work or Remaining at Home when Feeling Sick

	Agree or Strongly Agree				*P*-Value
	Totals	Students	Residents	Faculty	
*When feeling sick with what might be an infectious condition …*					
I would come into work to avoid creating more work for my colleagues (i.e., to avoid hassles related to coverage issues and letting my team down).	83%	83%	100%	71%	0.03
I would come into work to avoid negative repercussions (e.g., reprimand, disapproval).	60%	71%	84%	25%	< 0.001
I would come into work to avoid being seen as lazy or weak.	67%	75%	89%	40%	< 0.001
I feel a professional obligation to come into work no matter what.	31%	34%	42%	19%	0.16
*When feeling sick with what might be an infectious condition, physicians have a professional responsibility to …*					
Remain at home if they may not be able to function well because of their illness.	71%	67%	68%	81%	0.34
Remain at home to avoid spreading the illness to their patients.	81%	79%	74%	89%	0.32
Remain at home to limit spread of the illness to immuno-compromised patients.	78%	80%	78%	75%	0.82
Remain at home to avoid spreading the illness to their colleagues.	75%	73%	63%	83%	0.24
Come into work if they can use precautions to avoid spreading their illness (i.e. mask, gloves, etc.).	73%	76%	68%	69%	0.68

**Table 3:** Attitudes about Working When Feeling Sick, Specific to Training Level

	Agree or Strongly Agree		
	Students	Residents	Faculty
*As a medical student, when feeling sick with what might be an infectious condition …*			
I would leave the decision to come into work up to my superior.	33%	–	–
I would come into work to avoid a negative evaluation by my superior.	78%	–	–
I would come into work to avoid having to make up work.	64%	–	–
My decision to come into work would depend on the length of my rotation (i.e. I would be more likely to remain at home during a 6-week rotation as opposed to a 2-week rotation).	82%	–	–
*As a resident, when feeling sick with what might be an infectious condition …*			
I would leave the decision to come into work up to my superior.	–	37%	–
I would come into work to avoid a negative evaluation by my superior.	–	74%	–
*As a resident …*			
I would negatively evaluate a medical student for remaining at home when sick.	–	11%	–
I would view a medical student as lazy or weak if he or she called in sick.	–	5%	–
*As an attending (faculty) physician* …			
I would negatively evaluate a medical student or resident for remaining at home when sick with what might be an infectious condition.	–	–	0%
I would view a medical student or resident as lazy or weak if he or she called in sick.	–	–	3%

## Results

One hundred and twenty-seven participants completed the second survey (44% overall response rate), consisting of 69 medical students, 20 resident physicians, and 38 faculty physicians (with subgroup response rates of 49%, 42%, and 37%, respectively).

### Attitudes about working when sick, related to severity and type of illness

As shown in Table 1, a majority of participants (60%) affirmed a general sense of professional obligation to fulfill work responsibilities when they are sick, and this affirmation was more common in resident physicians and students than in faculty physicians (84% vs. 61% vs. 46%, *p* = 0.02). Participants’ attitudes toward work obligations varied according to the type and severity of illness: 95% felt an obligation to work with a non-febrile mild upper respiratory infection, 33% felt obliged to work with influenza-like symptoms (fever, myalgias, cough), and 13% reported they would work with a fever > 101.5 F. Resident physicians and medical students were more likely than faculty physicians to express an obligation to work with influenza-like symptoms (67% vs. 35% vs. 14%, *p* = 0.001). Substantial proportions of respondents reported they would go to work with illness that included vomiting (24%) or diarrhea (28%) within the last 8 h. In all of these categories of illness, resident physicians were the most likely to report an intention to work while feeling sick.

### Reasons for coming into work or remaining at home when feeling sick

Most participants endorsed a variety of reasons for why they would work when feeling sick with a potentially infectious condition, even as they also affirmed reasons for why physicians in general have a professional responsibility to remain at home when feeling sick with what might be an infectious disease. As shown in Table 2, large proportions of participants endorsed reasons for working when sick that expressed motivations to avoid creating more work for colleagues (83%), avoid negative repercussions for themselves (60%), and avoid appearing lazy or weak (67%). Residents and students were more likely than faculty physicians to want to avoid negative repercussions (84% vs 71% vs. 25%, *p* < 0.001) or appearing lazy or weak (89% vs 75% vs. 40%, *p* < 0.001). Nearly a third of participants endorsed the conviction that they have a professional obligation to “work no matter what” (31%). Resident physicians and students were more likely than faculty physicians to endorse these reasons.

As Table 2 further demonstrates, a majority of participants also recognized a number of reasons for the general professional responsibility physicians have to remain at home when they feel sick with what might be an infectious disease. These reasons focused on the need to be able to function well at work (71%) and to avoid spreading infections to patients (81%), immunocompromised patients (78%), or colleagues (75%). However, these reasons were qualified by the belief that physicians have a professional responsibility to work while feeling sick with a potentially infectious disease if precautions can be used to avoid the spread of infection (73%).

### Additional attitudes about working when feeling sick, specific to training level

Table 3 shows that only a minority of medical students (33%) and resident physicians (37%) would rely on their superiors to decide whether they (the student or resident) should work when feeling sick with a potentially infectious condition, and most students (78%) and residents (74%) would be motivated to work when feeling sick in order to avoid a negative evaluation by a superior. Many students also indicated a desire to fulfill their work obligations in order to avoid the burden of make up work (64%), and they were more likely to be inclined to work if they were on a shorter rotation (82%). Regarding attitudes among supervising residents and faculty physicians toward students and trainees, few residents (11%), and no faculty physicians, indicated they would negatively evaluate a student/trainee for remaining at home when feeling sick with a potentially infectious condition, and very few residents (5%) and faculty physicians (3%) would view a student/trainee as lazy or weak if he or she called in sick.

## Discussion

The results of our study suggest that many medical students, resident physicians, and faculty physicians feel a professional obligation to fulfill work responsibilities even when they feel sick. This sense of duty is often motivated by a desire to avoid burdening colleagues with more work, avoid appearing lazy or weak, and avoid negative repercussions for themselves. All three of these motivations appear to be more common in residents and students than among faculty. Our results describe a willingness to work when sick that is consistent with previous studies of healthcare professionals in terms of general prevalence [8–17], and in terms of specific motivations to avoid burdening colleagues with more work [8, 9, 11, 16, 17], avoid appearing lazy [17], avoid negative repercussions [16], avoid having to make up work [8, 9, 17], in addition to an implicit or stated sense of duty to patients [8, 16, 17].

We did not expect to find, however, that one third of our participants felt obligated to work with influenza-like symptoms (fever, myalgias, cough), that approximately a quarter of our participants would work despite having had vomiting or diarrhea within the last 8 h, and that 13% reported they would work with a fever > 101.5 F. These data suggest that a substantial proportion of residents and students (and some faculty) are so motivated to fulfill their work responsibilities that knowledge of the potential infectiousness of a condition is not by itself enough of a reason to prevent them from working with patients.

Our study’s focus on symptoms of influenza deserves careful attention, since influenza can serve as a paradigm case to assess attitudes toward sickness presenteeism among healthcare professionals in light of established clinical and epidemiological knowledge and guidelines for prevention. The CDC guidelines are clear: healthcare professionals who have fever and respiratory symptoms should not work with patients and should be excluded from work until they are afebrile for at least 24 h [26]. Despite such clear recommendations, there is evidence to suggest that over 40% of healthcare professionals with influenza-like illness work when they are sick [11, 12]. Though influenza virus is not the only potential microbe that healthcare professionals might transmit to their patients [23, 27, 28], it is concerning that the hallmark symptoms of such a well-known virus are not sufficient to keep some healthcare professionals from working with patients, despite widespread efforts to reduce the risk of influenza transmission in hospitals through behavioral measures and vaccination programs aimed at promoting patient safety [29, 30].

The results of our study demonstrate an ethical tension between an obligation to work and an obligation not to harm. Most of our study participants affirmed a variety of reasons for why they would work when feeling sick with a potentially infectious condition, even as they also affirmed reasons for why physicians in general have a professional responsibility to remain at home when feeling sick with what might be an infectious disease. These reasons to stay home focused on the need to be able to function well and to avoid spreading infections to patients or colleagues. However, these cautions about avoiding harm to patients and colleagues were qualified by the prevalent belief that if precautions can be used to avoid the spread of infection, physicians have a professional responsibility to work while feeling sick with a potentially infectious disease. The inclination to use such precautions in sickness presenteeism can be seen as an attempt to resolve the ethical tension by reconciling the duty to care with the duty not to harm [8, 10, 16]. The question remains whether such attempts are ethically justifiable and scientifically sound in the given circumstances of a particular case.

Our study included a particular focus on the attitudes of medical students and resident physicians with respect to their status as trainees, and several differences between these trainees and faculty physicians were observed. First, the general sense of professional obligation to fulfill work responsibilities when sick was more common in residents and students than in faculty. Second, residents and students were more likely than faculty to express an intention to work with influenza-like symptoms. Third, and perhaps most concerning in relation to training pressures, residents and students were more likely than faculty to want to avoid negative repercussions or appear lazy or weak, and most students and residents would be motivated to work when feeling sick in order to avoid a negative evaluation by a superior. These last findings reflect a perceived need to meet others’ expectations despite risks to patients and burdens to oneself. The frequencies of these attitudes in our study were higher than has been reported by other authors: 20% of students had concerns about evaluations in the study by Veale et al. [19] and 12% of residents were concerned about appearing weak in the report by Jena et al. [21] We also note with concern that only a minority of students and residents in our study indicated they would seek guidance from their superiors to decide whether they should work when feeling sick with a potentially infectious condition. This lack of deference to those whose professional responsibilities include both patient and trainee welfare may further reflect a desire to avoid negative repercussions. It also suggests an unwillingness to share accountability for an important decision – a problem that has also been observed outside of training environments (59% of clinical staff in a New Zealand hospital did not seek professional advice about sickness presenteeism decisions) [9].

Our study had limitations. First, although the survey was anonymous, social desirability bias may have led some participants to give answers that were perceived to be more socially acceptable. Second, answers to hypothetically-phrased questions may not predict actual behavior. Third, the absence of information from non-respondents raises the possibility of bias in our sample due to a response rate of 44%. Fourth, because our surveys were performed in 2012–13, there is the possibility our data may not represent current attitudes; however, this possibility seems unlikely, given that the most recent studies on sickness presenteeism (whose data were collected between 2014 and 2015) have continued to show similar prevalence rates (41%–88%) and motivations [8, 10, 11, 16]. Fifth, except for questions about “a cold” and “the flu”, we did not query participants’ attitudes toward working when ill with other specific infectious diseases whose greater or lower risks of transmission might have led to additional insights into the impact of transmission risk on decision making. Lastly, our participants were based in a single academic medical center in the US, and the resident and faculty physicians were proportionately fewer in number and represented only three specialties (Internal Medicine, Pediatrics, and Family Medicine), so our results may not be generalizable to physicians in other specialties, other practice settings, or other countries. Regarding the potential for specialty-based differences, although one study of resident physicians found no difference in rates of sickness presenteeism among different specialties (surgery, obstetrics/gynecology, internal medicine, pediatrics) [20], another study showed that surgeons were particularly sensitive to the impact on patients when procedures have to be rescheduled because of illness among surgeons and that both surgical residents and staff were more likely (than internal medicine colleagues) to report sickness presenteeism and less likely to be pleased when a sick colleague stayed at home [18].

## Conclusions

Sickness presenteeism represents a significant challenge. The factors that motivate it form a constellation of concerns that reflect the intersecting and in some ways competing interests of patients, colleagues, and sick healthcare professionals themselves. From the vantage point of the sick healthcare professional, these interests may at times be overlapping and mutually reinforcing (e.g., when staying home is simultaneously good for a sick healthcare professional and for his/her patients). At other times they may be competing and in tension (e.g., when staying home is good for a sick healthcare professional and for his/her patients, but creates more work for colleagues). In the real world of healthcare, there will be numerous clinical and practical details of the actual circumstances at hand that need to be considered to determine whether, in a specific case, it is best for a sick healthcare professional to stay at home or go to work. In some cases it should be obvious that staying at home is the right answer, but in many cases careful deliberation is needed. This process of deliberation requires practical wisdom that is facilitated by humility and dialogue [31] and guided by ethical priorities that make the health and safety of patients our overriding concern [32].

Sickness presenteeism also represents an important opportunity, especially in training environments, to demonstrate how multiple interests and responsibilities can be identified and prioritized in the practice of medicine. Patient safety and quality are central to patient care and should not be compromised for administrative efficiency, undue pressure from colleagues, fear of professional repercussions, or a misplaced sense of pride or dedication that risks putting personal endurance before patient welfare. To the extent that sickness presenteeism does arise from such motivations, it would be fair to ask whether sickness presenteeism participates in a hidden curriculum [33], one that quietly caters to a professionally oriented culture of efficiency and achievement rather than a patient-centered culture of service and safety. The data we report arguably support the conclusion that there is a hidden curriculum that encourages sickness presenteeism – especially among residents, who are further from the formal curriculum they were taught in medical school and more deeply embedded in, and reliant on, an intense work culture that includes peer expectations to persevere and avoid creating more work for colleagues.

Essential for a balanced assessment of sickness presenteeism is also a concern for the welfare of healthcare professionals themselves, especially trainees who may feel disempowered and vulnerable. If sickness presenteeism is part of a hidden curriculum, addressing it will require a change in attitude and culture [10, 34] so that hospitals, clinics, training programs, and medical schools communicate the implications of sickness presenteeism for patient safety [11, 27], educate new students and residents about CDC guidelines that prohibit working with patients when healthcare professionals have fever and respiratory symptoms [26], and provide the means for accommodation and coverage when healthcare professionals are sick [18, 35]. Changes may also need to include impressing upon trainees the importance of seeking counsel from supervisors so that decisions about sickness presenteeism are properly informed and responsibly shared. Such changes in attitude and culture would promote health for patients and professionals alike, and they would resonate deeply with the ancient call to “first do no harm.”

## Data Availability

All data generated or analyzed during this study are included in this published article.

## Appendix

**Table 4:** Codes, frequencies by participant group, and inter-rater reliability

Code	Students		Residents		Faculty		Kappa Score
	*#*	*(%)*	*#*	*(%)*	*#*	*(%)*	
Transmission risk/Direct patient care responsibilities	21	(21.9)	10	(22.2)	17	(29.8)	0.853
Severity of illness	22	(22.9)	7	(15.6)	12	(21.1)	0.909
Creating more work for colleagues, including hassles related to coverage issues and letting “my team” down	5	(5.2)	7	(15.6)	10	(17.5)	0.794
Fever	9	(9.4)	7	(15.6)	4	(7.0)	0.938
Ability to perform work and make decisions well	11	(11.5)	1	(2.2)	5	(8.8)	0.932
How will calling in sick look to my superior	9	(9.4)	2	(4.4)	0	(0.0)	1.000
Obligation to work no matter what	1	(1.0)	5	(11.1)	0	(0.0)	0.600
Avoiding reprimand, disapproval or other negative repercussions	2	(2.1)	3	(6.7)	1	(1.8)	1.000
Vomiting	1	(1.0)	2	(4.4)	2	(3.5)	0.905
Diarrhea	1	(1.0)	1	(2.2)	2	(3.5)	1.000
Type of patient	3	(3.1)	0	(0.0)	1	(1.8)	0.853
Avoid having to make up work	4	(4.2)	0	(0.0)	0	(0.0)	0.796
Direct contact with/threat to other staff	1	(1.0)	0	(0.0)	2	(3.5)	0.796
Type of rotation	3	(3.1)	0	(0.0)	0	(0.0)	0.392
Ability to use precautions	1	(1.0)	0	(0.0)	1	(1.8)	1.000
Desire to go to work	1	(1.0)	0	(0.0)	0	(0.0)	1.000
Other	1	(1.0)	0	(0.0)	0	(0.0)	1.000
*Totals*	*96*	*(100.0)*	*45*	*(100.0)*	*57*	*(100.0)*	*–*

## References

[1] Aronsson G, Gustafsson K, Dallner M (2000). Sick but yet at work. An empirical study of sickness presenteeism. J Epidemiol Community Health.

[2] Hansen CD, Andersen JH (2008). Going ill to work – what personal circumstances, attitudes and work-related factors are associated with sickness presenteeism?. Soc Sci Med.

[3] Rainbow JG, Steege LM (2017). Presenteeism in nursing: an evolutionary concept analysis. Nurs Outlook.

[4] Schultz AB, Edington DW (2007). Employee health and presenteeism: a systematic review. J Occup Rehabil.

[5] Schultz AB, Chen C-Y, Edington DW (2009). The cost and impact of health conditions on presenteeism to employers: a review of the literature. Pharmacoeconomics..

[6] Lack DM (2011). Presenteeism revisited: a comprehensive review. AAOHN J.

[7] Skagen K, Collins AM (2016). The consequences of sickness presenteeism on health and wellbeing over time: a systematic review. Soc Sci Med.

[8] Al Nuhait M, Al Harbi K, Al Jarboa A, Bustami R, Alharbi S, Masud N, Albekairy A, Almodaimegh H (2017). Sickness presenteeism among health care providers in an academic tertiary care center in Riyadh. J Infect Public Health.

[9] Bracewell LM, Campbell DI, Faure PR, Giblin ER, Morris TA, Satterthwaite LB, Simmers CDA, Ulrich CM, Holmes JD (2010). Sickness presenteeism in a New Zealand hospital. NZ Med J.

[10] Chambers C, Frampton C, Barclay M (2017). Presenteeism in the New Zealand senior medical workforce—a mixed methods analysis. NZ Med J..

[11] Chiu S, Black CL, Yue X, Greby SM, Laney AS, Campbell AP, de Perio MA (2017). Working with influenza-like illness: Presenteeism among US health care personnel during the 2014-2015 influenza season. Am J Infect Control.

[12] Esbenshade JC, Edwards KM, Esbenshade AJ, Rodriguez VE, Talbot HK, Joseph MF (2013). Respiratory virus shedding in a cohort of on-duty healthcare workers undergoing prospective surveillance. Infect Control Hosp Epidemiol.

[13] Rosvold EO, Bjertness E (2001). Physicians who do not take sick leave: hazardous heroes?. Scand J Public Health.

[14] Sendén MG, Løvseth LT, Schenck-Gustafsson K, Fridner A (2013). What makes physicians go to work while sick: a comparative study of sickness presenteeism in four European countries (HOUPE). Swiss Med Wkly.

[15] Sendén MG, Schenck-Gustafsson K, Fridner A (2016). Gender differences in reasons for sickness presenteeism - a study among GPs in a Swedish health care organization. Ann Occup Environ Med.

[16] Szymczak JE, Smathers S, Hoegg C, Klieger S, Coffin SE, Sammons JS (2015). Reasons why physicians and advanced practice clinicians work while sick: a mixed-methods analysis. JAMA Pediatr.

[17] Tan PC, Robinson G, Jayathissa S, Weatherall M (2014). Coming to work sick: a survey of hospital doctors in New Zealand. NZ Med J..

[18] Gudgeon P, Wells DA, Baerlocher MO, Detsky AS (2009). Do you come to work with a respiratory infection?. Occup Environ Med.

[19] Veale PM, Vayalumkal JV, McLaughlin K (2016). Sickness presenteeism in clinical clerks: negatively reinforced behavior or an issue of patient safety?. Am J Infect Control.

[20] Jena AB, Baldwin DC, Daugherty SR, Meltzer DO, Arora VM (2010). Presenteeism among resident physicians. JAMA..

[21] Jena AB, Meltzer DO, Press VG, Arora VM (2012). Why physicians work when sick. Arch Intern Med.

[22] Mitchell KJ, Vayalumkal JV (2017). Sickness presenteeism: the prevalence of coming to work while ill among paediatric resident physicians in Canada. Paediatrics Child Health.

[23] Huttunen R, Syrjänen J (2014). Healthcare workers as vectors of infectious diseases. Eur J Clin Microbiol Infect Dis.

[24] ABIM Foundation (2002). American Board of Internal Medicine. Medical professionalism in the new millennium: a physician charter. Ann Intern Med.

[25] Hsieh HF, Shannon SE (2005). Three approaches to qualitative content analysis. Qual Health Res.

[26] Centers for Disease Control and Prevention. Prevention Strategies for Seasonal Influenza in Healthcare Settings. https://www.cdc.gov/flu/professionals/infectioncontrol/healthcaresettings.htm. Accessed 29 May 2019.

[27] Widera E, Chang A, Chen HL (2010). Presenteeism: a public health hazard. J Gen Intern Med.

[28] Kobayashi M, Lyman MM, Francois Watkins LK, Toews K-A, Bullard L, Radcliffe RA, Beall B, Langley G, Van Beneden C, Stone ND (2016). A cluster of group a streptococcal infections in a skilled nursing facility—the potential role of healthcare worker Presenteeism. J Am Geriatr Soc.

[29] Ahmed F, Lindley MC, Allred N, Weinbaum CM, Grohskopf L (2014). Effect of influenza vaccination of healthcare personnel on morbidity and mortality among patients: systematic review and grading of evidence. Clin Infect Dis.

[30] Payet C, Voirin N, Ecochard R, Vanhems P (2016). Influence of observable and unobservable exposure on the patient’s risk of acquiring influenza-like illness at hospital. Epidemiol Infect.

[31] Kaldjian LC (2014). Practicing medicine and ethics: integrating wisdom, conscience, and goals of care.

[32] Greiner AM, Kaldjian LC (2018). Rethinking medical oaths using the physician charter and ethical virtues. Med Educ.

[33] Hafferty FW, Franks R (1994). The hidden curriculum, ethics teaching, and the structure of medical education. Acad Med.

[34] Thompson WT, Cupples ME, Sibbett CH, Skan DI, Bradley T (2001). Challenge of culture, conscience, and contract to general practitioners' care of their own health: qualitative study. BMJ..

[35] Landry M, Miller C (2010). Presenteeism: are we hurting the patients we are trying to help?. J Gen Intern Med.

